# Gene and pathway identification with *L*_*p *_penalized Bayesian logistic regression

**DOI:** 10.1186/1471-2105-9-412

**Published:** 2008-10-03

**Authors:** Zhenqiu Liu, Ronald B Gartenhaus, Ming Tan, Feng Jiang, Xiaoli Jiao

**Affiliations:** 1 Division of Biostatistics, University of Maryland Greenebaum Cancer Center, 22 South Greene Street, Baltimore, MD 21201, USA; 2 Department of Medicine and Greenebaum Cancer Center, The University of Maryland School of Medicine, Baltimore, MD 21201, USA; 3 Department of Pathology, The University of Maryland School of Medicine, Balitimore, MD 21201, USA

## Abstract

**Background:**

Identifying genes and pathways associated with diseases such as cancer has been a subject of considerable research in recent years in the area of bioinformatics and computational biology. It has been demonstrated that the magnitude of differential expression does not necessarily indicate biological significance. Even a very small change in the expression of particular gene may have dramatic physiological consequences if the protein encoded by this gene plays a catalytic role in a specific cell function. Moreover, highly correlated genes may function together on the same pathway biologically. Finally, in sparse logistic regression with *L*_*p *_(*p *< 1) penalty, the degree of the sparsity obtained is determined by the value of the regularization parameter. Usually this parameter must be carefully tuned through cross-validation, which is time consuming.

**Results:**

In this paper, we proposed a simple Bayesian approach to integrate the regularization parameter out analytically using a new prior. Therefore, there is no longer a need for parameter selection, as it is eliminated entirely from the model. The proposed algorithm (BLpLog) is typically two or three orders of magnitude faster than the original algorithm and free from bias in performance estimation. We also define a novel similarity measure and develop an integrated algorithm to hunt the regulatory genes with low expression changes but having high correlation with the selected genes. Pathways of those correlated genes were identified with DAVID .

**Conclusion:**

Experimental results with gene expression data demonstrate that the proposed methods can be utilized to identify important genes and pathways that are related to cancer and build a parsimonious model for future patient predictions.

## Background

Gene selection and cancer prediction with microarray data have been studied extensively in recent years. Most earlier studies concentrated on identifying a small number of discriminatory genes with different statistical and machine learning methods [1-3]. Many statistical learning techniques such as support vector machines [4], the relevance vector machines (RVM) [5,6], LASSO [7-9], and sparse logistic regression [10-12] have been applied to this problem. There are two common goals for such algorithms: The first is to distinguish cancer and non-cancer patients with the highest possible accuracy. The second is to identify a small subset of genes that are highly differentiated in different classes and to associate gene expression patterns with disease status. The genes identified with the second aim may improve our understanding of the underlying causes of the cancer. In gene selection, when genes share the same biological pathway, the correlation between them can be high [13], and those genes form a group. The ideal gene selection methods eliminate the trivial genes and automatically include the whole group genes into the model once one gene among them is selected. Most importantly, almost all of the current methods are biased towards selecting those genes that display the most pronounced expression differences. Such methods select genes using purely statistical criteria (either rank score or classification accuracy) and this selection is thought to reflect their relative importance. Quite often, a certain number of genes with the smallest p-values or highest prediction accuracy are finally selected, while most biologists recognize that the magnitude of differential expression does not necessarily indicate biological significance. From the biological prospective, even a very small change in the expression of a particular gene may have dramatic physiological consequences if the protein encoded by this gene plays a catalytic role in a specific cell function [14]. Many other downstream genes may amplify the signal produced by this truly interesting gene, thereby increasing their chance of being selected by current gene selection methods. For a regulatory gene, however, the chance of being selected by such methods may diminish as one keeps hunting for downstream genes that tend to show much bigger changes in their expression. As a result, the initial list of candidate genes may be enriched with many effector genes that do little to elucidate more fundamental mechanisms of biological processes. Therefore we have to deal with two important problems in gene selection: (1) how to take into account the gene-gene correlations and (2) how to hunt the upstream regulatory genes. The characteristic of the regulatory genes is that their gene expression changes may be low, but they are highly correlated with the downstream highly expressed genes. Although there is ongoing research to incorporate prior biological knowledge, such as partially known pathways in gene selection [15], to the best of our knowledge, there is no efficient method to hunt the upstream regulatory genes in gene selection and pathway discovery. There is, therefore, a pressing need for new algorithms to be developed.

In this paper, we propose a substantial improvement to the sparse logistic regression (SparseLOGREG) approach [12]. The SparseLOGREG algorithm employs a *L*_*p *_norm regularization [16], which is equivalent to super Laplace prior over the model parameters. Both the generalization ability of the model and the sparsity achieved are critically dependent on the value of a regularized parameter, which has to be carefully tuned to the best performance. This best parameter can only be found through cross-validation and computationally intensive search. In this paper, the regularization parameter, however, will be integrated out analytically using a new prior that is similar to the uninformative Jeffery's prior [11]. The resulting algorithm (BLpLog) has the comparable performance with the original algorithm but is much faster, as there is no longer a need for parameter optimization. The goal of the current study is to develop a computationally affordable and well-behaved estimating approach, which can effectively identify cancer related genes and pathways. We propose an integrated method that first identifies a small subset of cancer related genes utilizing the *L*_*p *_regularized Bayesian logistic regression (BLpLog), and then define a novel similarity measure to identify the regularized genes that are highly correlated with each gene in the subset. Finally, we annotate the regularized genes and identify the cancer related pathways using DAVID.

## Results and discussion

In this section, we evaluate the performance of proposed *L*_*p *_penalized Bayesian logistic regression (BLpLog) methods and the integrated algorithm using several microarry data. We compare proposed method with SparseLOGREG [12] and BLogReg [11].

SparseLOGREG includes a regularization parameter, controlling the complexity of the model and the sparsity of the model parameters, which must be chosen by the user or alternatively optimized in an additional model selection stage. Therefore, the value of this parameter is found via a (computationally expensive) maximization of the cross-validation estimate of the area under the ROC curve (AUC). However, we cannot use the same cross-validation estimate for both model selection and performance evaluation as this would introduce a strong selection bias in favor of the existing sparse SparseLOGREG model. A nested cross-validation procedure is therefore used instead. 10-fold cross validation is used for performance evaluation in the 'outer loop' of the procedure, in each iteration of which model selection is performed individually for each classifier based on a separate leave-one-out cross-validation procedure using the training data only. Because of the small sample size and high dimensional genes, leave-one-out cross validation in the 'inner loop' likely provide a reliable performance measure for model selection. Even though this nested cross-validation is computationally expensive, it provides an almost unbiased assessment of generalization performance as well as a sensible automatic method of setting the value of the regularization parameter. We do not need model selection with both BLpLog and BLogReg, only 10-fold cross validation is used for performance evaluation. Finally, we find the highly correlated upstream genes with proposed correlation measure and identify the related pathways using DAVID.

### Breast Cancer Data Set [17]

98 primary breast cancers (34 from patients who developed distant metastases within 5 years, 44 from patients who continue to be disease-free after a period of at least 5 years, 18 from patients with BRCA1 germline mutations and 2 from BRCA2 carriers) have been selected from patients who were lymph node negative and under 55 years of age at diagnosis. There is a total of 24188 genes. This data set contained some missing values. Gene expression levels lacking for all patients are left out. The rest of the missing values are estimated based on the correlations between gene expressions.

**Figure 1:**
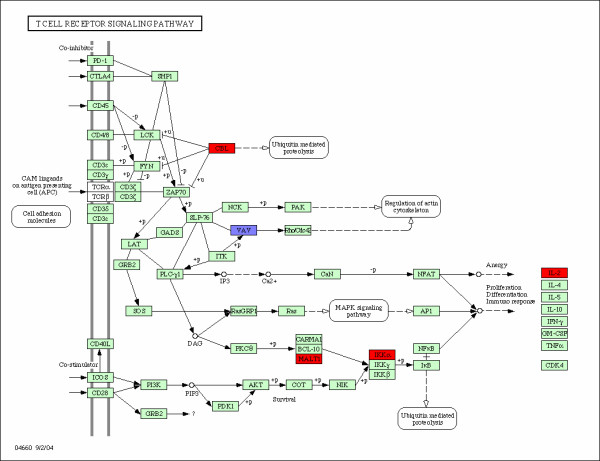
**T Cell Receiptor Signaling Pathway**. T-cell receipt signaling pathway and the associated genes identified with the integrated algorithm. The over-expressed and down-expressed genes on the pathway are shown in red and blue respectively.

**Figure 2:**
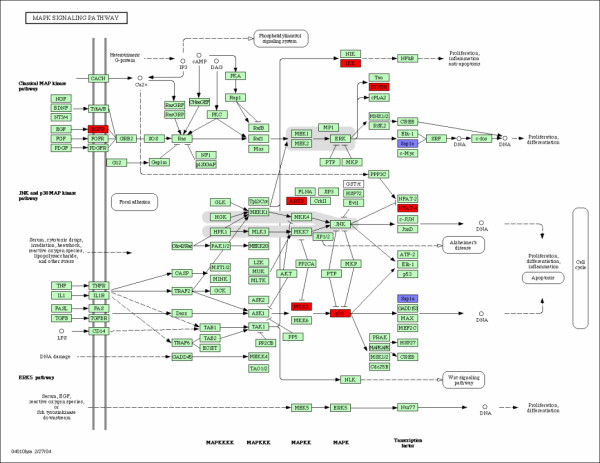
**MAPK Signaling Pathway**. MAPK signaling pathway and the associated genes. The over-expressed and down-expressed genes on the pathway are shown in red and blue respectively.

We apply the proposed integrated algorithm to the data. BLpLog identifies 11 genes with the 10-cross-validation *AUC *= 0.976. These 11 genes are highly differentiated in patients with and without metastases. SparseLOGREG selects 10 highly differentiated genes with predicted *AUC *= 0.981, and BLogReg identifies 14 genes with the predicted *AUC *= 0.953. Both SparseLOGREG and BLpLog outperform BLogReg with higher AUC value and less genes. but that the difference in performance between the SparseLOGREG and BLpReg algorithms is minimal. The BLpLog algorithm is marginally more computational expensive than the BLogReg with multiple initializations. It takes 5 minutes compare 1.7 minutes with BLogReg algorithm. The SparseLOGREG algorithm is very much more expensive, owing to the need for a model selection stage to choose a good value for the regularization parameter. It takes roughly 4 hours on the same PC. Given the minimal difference in performance and substantial difference in computational expense there is little reason to prefer the SparseLOGREG over the BLpLog algorithm. We find the correlated genes for each of the 11 selected genes using the criteria |*R*| > 0.9 and identify pathways that associated with those genes with DAVID. The 11 genes with BLpLog are listed in Table 1. Each pathway is identified in such a way that the statistical significance of the pathway is the highest (p value is the smallest) in DAVID. The '+' and '-' signs in column 2 of Table 1 indicate that the selected gene is either over-expressed or down-expressed for patients with metastases. The total number of highly correlated genes with each selected gene is given in column 4. The highly correlated genes on a KEGG pathway are shown in Table 2. Six pathways associated with 7 selected genes are identified. The correlated genes of the 4 other selected do not have a KEGG pathway associated with them. The plots of T cell receiptor signaling and MAPK signaling pathway are shown in Figure 1 – Figure 2. The over-expressed and and down-expressed genes on the pathway are shown in red and blue respectively. Each of these six pathways plays an important role in breast cancer survivals. For instance, JAK/STAT signaling pathway is the principal signaling mechanism for a wide array of cytokines and growth factors. JAK activation stimulates cell proliferation, differentiation, cell migration and apoptosis. These cellular events are critical to hematopoiesis, immune development, mammary gland development and lactation, adipogenesis, sexually dimorphic growth and other processes. Predictably, mutations that reduce JAK/STAT pathway activity affect these processes. Conversely, mutations that constitutively activate or fail to regulate JAK signaling properly cause inflammatory disease, erythrocytosis, gigantism and different cancers. Moreover, LEUKOCYTE TRANSENDOTHELIAL MIGRATION provides relevant information about how cells interact with the endothelium and transmigrate. Transendothelial migration of cancer cells from the vasculature into tissue stroma is a final step in the metastatic cascade, prior to formation of secondary tumors. Patients who developed distant metastases in less than 5 years and those who had no distant metastases have 8 genes differentially expressed. The proposed integrated algorithm provides information not only about the set of genes involved on these pathways, but also about how genes interact and regulate each other. In this manuscript, we will only discuss both T CELL RECEPTOR SIGNALING PATHWAY and MAPK SIGNALING PATHWAY in more detail. Other pathways can be analyzed in a similar fashion.

**Table 1:** BLpLog selected genes and the number of correlated

Gene ID	Gene Name	Gene Description	# of Corr. Genes.
Contig27800_RC	SFTPD (+)	surfactant, pulmonary associated protein D	213
NM_000909	STXBP1 (-)	syntaxin binding protein 1	411
NM_003147	(-)	ESTs	229
Contig23399_RC	(-)	ESTs	227
Contig38438_RC	(-)	ESTs	327
NM_003882	(-)	ESTs	110
AF221520	LOC51241 (+)	hypothetical protein	274
AL080059	TCP1 (+)	t-complex 1	169
U79298	ACTR2 (-)	ARP2 homolog	46
NM_001197	FLJ10375 (-)	hypothetical protein FLJ10375	10
NM_000599	ZNF83 (+)	hypothetical protein FLJ11015	30

**Table 2:** Highly correlated genes and KEGG pathway

Genes	Gene Description	Pathway
**STXBP1**	**NM_000909**	

CNTFR	ciliary neurotrophic factor receptor	JAK-STAT SIGNALING PATHWAY
PRL	prolactin	
SOCS3	suppressor of cytokine signaling 3	
EPOR	erythropoietin receptor	
JAK2	janus kinase 2	
CBL	cas-br-m (murine) ecotropic retroviral transforming sequence	
IL5RA	interleukin 5 receptor, alpha	

**ESTs**	**NM_003147**	

SEMA3E	sema domain, immunoglobulin domain, short basic domain, (semaphorin) 3e	AXON GUIDANCE
RASA1	ras p21 protein activator (gtpase activating protein) 1	
SLIT3	slit homolog 3 (drosophila)	
SRGAP3	slit-robo rho gtpase activating protein 3	
EFNA5	ephrin-a5	

**ESTs**	**Contig38438**	

CLDN14	claudin 14	LEUKOCYTE TRANSENDOTHELIAL MIGRATION
MAPK13	mitogen-activated protein kinase 13	
CLDN4	claudin 4	
RAP1B	member of ras oncogene family	
PTK2B	protein tyrosine kinase 2 beta	
CDC42	cell division cycle 42	
MYL6	myosin, light polypeptide 6, smooth muscle and non-muscle	
VCL	vinculin	

**ESTs + ACTR2**	**NM_003882 + U79298**	

STMN1	stathmin 1/oncoprotein 18	MAPK SIGNALING PATHWAY
MAPK13	mitogen-activated protein kinase 13	
MAP2K3	mitogen-activated protein kinase kinase 3	
NFATC4	nuclear factor of activated t-cells, cytoplasmic, calcineurin-dependent 4	
CACNA1H	calcium channel, voltage-dependent, alpha 1 h subunit	
ARRB2	arrestin, beta 2	
FGFR3	fibroblast growth factor receptor 3	
ELK4	elk4, ets-domain protein	
IKBKE	inhibitor of kappa light polypeptide gene enhancer in b-cells, kinase epsilon	

**LOC51241**	**AF221520**	

VAV2	vav 2 oncogene	T CELL RECEPTOR SIGNALING PATHWAY
IL2	interleukin 2	
CHUK	conserved helix-loop-helix ubiquitous kinase	
MALT1	mucosa associated lymphoid tissue lymphoma translocation gene 1	
CBL	cas-br-m (murine) ecotropic retroviral transforming sequence	

**TCP1**	**AL080059**	

COX10	cox10 homolog, cytochrome c oxidase assembly protein	OXIDATIVE PHOSPHORYLATION
ATP6V0A2	atpase, h+ transporting, lysosomal v0 subunit a2	
COX4I1	cytochrome c oxidase subunit iv isoform 1	
COX6B1	cytochrome c oxidase subunit vib polypeptide 1 (ubiquitous)	

T Cell Receptor (TCR) Signaling (Figure 1) induces activation of multiple tyrosine kinases, resulting in the phosphorylation of numerous intracellular substrates. One of the first steps in the generation of the immune response is the recognition by T lymphocytes of peptide fragments (antigens) derived from foreign pathogens that are presented on the surface of antigen presenting cells (APC). This event is mediated by the T cell receptor (TCR), which transduces these extracellular signals by initiating a wide array of intracellular signaling pathways. This signaling pathway is one of the identified targets for breast cancer drug development. We identified 5 genes on the pathway: CBL, VAV, MALT1, CHUK (IKK*α*), and IL-2. VAV2, MALT1, and CHUK(IKK*α*) are also on the B-cell receiptor signal pathway. Among them, only gene VAV2 is down-expressed in patients with distant metastases in less than 5 years. The other 4 genes are up-expressed. VAV2 is an oncogene and plays a critical role in hematopoietic signal transduction. The down-expressed Vav2 has been implicated in breast cancer metastasis and may prove to be very important in the aberrant activation of Rho GTPases during the metastatic cascade. The other 4 over-expressed genes are also very important for breast and other cancers and were well studied in the literature. For example, the CBL oncogene was first identified as part of a transforming retrovirus which induces mouse pre-B and pro-B cell lymphomas. As an adaptor protein for receptor protein-tyrosine kinases, it positively regulates receptor protein-tyrosine kinase ubiquitination in a manner dependent upon its variant SH2 and RING finger domains. Ubiquitination of receptor protein-tyrosine kinases terminates signaling by marking active receptors for degradation. MALT1, CHUK, and IL-2 are also important oncogene identified. These 3 genes have the causal relations as shown in Figure 1. MALT1 is defined as the mucosa associated lymphoid tissue lymphoma translocation gene 1. The over-expressed MALT2 in patients with distant metastasis causes the over-expressed CHUK, and then the over-expressed IL-2. Therefore, MALT1 is essential for T cell activation, proliferation, and IL-2 production. If MALT1 is not present, both CHUK and IL-2 will shut-off.

The Mitogen-Activated Protein Kinase (MAPK) signaling pathway (Figure 2) transduces a large variety of external signals, leading to a wide range of cellular responses, including growth, differentiation, inflammation and apoptosis. The MAPK signaling pathway has been linked to being responsible for the malignant phenotype, including increased proliferation, defects in apoptosis, invasiveness and ability to induce neovascularization. Consequently, different therapies towards inhibiting the pathway are under development. Nine genes were identified on the pathway. Patients with metastases in less than 5 years are over-expressed in 8 genes and down-expressed in one gene (Table 2 and Figure 2).

There are several causal relations among them. For instance, EGFR belong to the family of epidermal growth factor receptors and has been proven to play major roles in different histological types of breast cancer. The over-expressed EGFR in patients with metastases may be responsible for the up-expressed STMN1 and the down-expressed ELK4 (Sap1a). ELK4 is a downstream gene on the MAPK pathway. Moreover, the the over-expressed MAP2K3 (MKK3) causes the over-expressed MAPK12(P38), and then causes the down-expressed ELK4 (Sap1a). The systematic review of the interactions among the correlated genes on a specific pathway provides us more information about how various genes interact with each other and which gene plays a catalytic role and is more important. EGFR3 is certainly a more important upstream gene and mutations that lead to EGFR over-expression (or overactivity) have been associated with a number of cancers. The over-expressed EGFR in patients with metastases has led to the development of anticancer therapeutics directed against EGFR.

### Hepatocellular carcinoma data set [18]

mRNA expression profiles in tissue specimens from 60 hepatocellular carcinoma tissues of which 20 suffer from early intrahepatic recurrence and 40 do not. The number of gene expression levels is 7129. Since hepatocellular carcinoma has a poor prognosis because of the high intrahepatic recurrence rate, the original goal is to predict early intrahepatic recurrence or non-recurrence. With the proposed integrated algorithm, we can identify not only the highly differentiated genes but also the related pathways.

**Figure 3:**
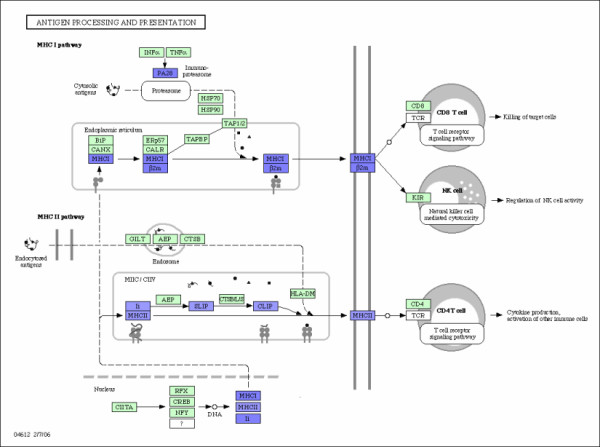
**Antigen Processing and Presentation**. Antigen processing and presentation pathway and the associated genes. The over-expressed and down-expressed genes on the pathway are shown in red and blue respectively.

**Figure 4:**
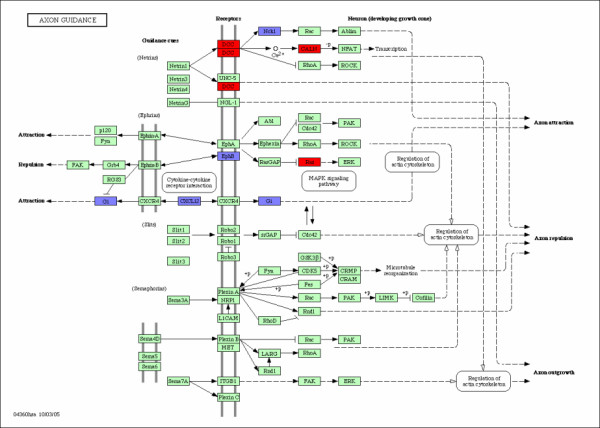
**Axon Guidance**. Axon guidance pathway and the associated genes. The over-expressed and down-expressed genes on the pathway are shown in red and blue respectively.

BLpLog identifies 8 highly differentiated genes with the test *AUC *= 0.93 with the computational time of 3.2 minutes. BLogReg selects 13 genes with the predicted *AUC *= 0.90 and computational time of 1.6 minutes. SparseLOGREG identifies 10 genes with the predicted *AUC *= 0.936 and computational time of 127 minutes (2 hours). The selected genes with different methods are not completely the same but highly correlated. Again with the minimal difference in performance and big differences in computational time between BLpLog and SparseLOGREG, obviously BLpLog is preferred. The highly correlated genes and corresponding pathways are selected with the integrated algorithm. Table 3 and Table 4 are the computational results. Seven pathways was identified from the data. The plots of Antigen Processing and Presentation and Axon Guidance pathways are shown in Figure 3 – Figure 4. All eight pathways identified are important in hepatocellular carcinoma and other cancers. For example, PURINE METABOLISM pathway is one of the metabolism pathways involved in nucleotide synthesis and degradation, amino acid catabolism, non-essential amino acid synthesis and the urea cycle. Understanding the mechanism involved in metabolic regulation has important implications in both biotechnology and medicine. It is estimated that at least a third of all serious health problems are caused by metabolic disorders. Analyzing differentiated expressed genes on the pathway may provide some insight on the early intrahepatic recurrence of hepatocellular carcinoma after curative resection. Other pathways such as CYTOKINE-CYTOKINE RECEPTOR INTERACTION, NEUROACTIVE LIGAND-RECEPTOR INTERACTION, MAPK SIGNALING PATHWAY, and GAP JUNCTION are all hepatocellular carcinoma related. We will discuss the two pathways ANTIGEN PROCESSING AND PRESENTATION and AXON GUIDANCE in more details.

**Table 3:** BLpLog selected genes and the number of correlated

A3y ID	Gene Description	# of Corr. Genes
D26600_at (+)	Human mRNA for proteasome subunit HsN3	133
M16973_at (-)	Human complement protein C8 beta subunit mRNA	124
M63573_at (+)	Human secreted cyclophilin-like protein (SCYLP) mRNA	103
U79294_at(-)	Human clone 23748 mRNA, complete cds	102
U94586_at(+)	ubiquinone oxidoreductase MLRQ subunit mRNA	122
X00274_at(-)	Human gene for HLA-DR alpha heavy chain a class II antigen (immune response gene) of the MHC	92
X59798_at (+)	Human PRAD1 mRNA for cyclin	107
X69141_at (+)	H. sapiens mRNA for squalene synthase	99

**Table 4:** KEGG pathway and the highly correlated genes

Genes	Gene Description	Pathway
**D26600_at and X59798_at**		

X06562_at	growth hormone receptor	CYTOKINE-CYTOKINE RECEPTOR
X95095_at	platelet-derived growth factor receptor, alpha polypeptide	
M12783_at	platelet-derived growth factor beta polypeptide (simian sarcoma viral (v-sis) oncogene homolog)	
M20137_at	interleukin 3 (colony-stimulating factor, multiple)	
L36033_at	chemokine (c-x-c motif) ligand 12 (stromal cell-derived factor 1)	
V00532_rna1_f_at	interferon, alpha 17	
U11877_at	interleukin 8 receptor, beta	
V00542_f_at	interferon, alpha 14	
D49410_at	interleukin 3 receptor, alpha (low affinity)	
X04688_at	interleukin 5 (colony-stimulating factor, eosinophil)	
X02958_at	interferon, alpha 6	
X00695_s_at	interleukin 2	
U02687_at	fms-related tyrosine kinase 3	
M83554_at	tumor necrosis factor receptor superfamily, member 8	
D43767_at	chemokine (c-c motif) ligand 17	
X02958_at	interferon, alpha 1	
U83326_s_at	chemokine (c-c motif) receptor 5	
X02958_at	interferon, alpha 5	

**M16973_at**		

M86868_at	gamma-aminobutyric acid (gaba) receptor, rho 2	NEUROACTIVE LIGAND RECEPTOR INTERACTION
U49395_at	purinergic receptor p2x, ligand-gated ion channel, 5	
U68133_at	cannabinoid receptor 1 (brain)	
K01911_at	neuropeptide y	
M31210_at	endothelial di3erentiation, sphingolipid g-protein-coupled receptor, 1	
D85376_at	thyrotropin-releasing hormone receptor	
M18185_at	gastric inhibitory polypeptide	
L27080_at	melanocortin 5 receptor	
X82068_at	glutamate receptor, ionotrophic, ampa 3	

**M63573_at**		

U61538_at	calcium binding protein p22	AXON GUIDANCE
M14949_at	related ras viral (r-ras) oncogene homolog	
L36033_at	chemokine (c-x-c motif) ligand 12 (stromal cell-derived factor 1)	
X17576_at	nck adaptor protein 1	
M17219_at	guanine nucleotide binding protein (g protein), alpha inhibiting activity polypeptide 1	
L41939_at	eph receptor b2	
X76132_at	deleted in colorectal carcinoma	

**U79294**		

M92432_at	guanylate cyclase 2d, membrane (retina-specific)	PURINE METABOLISM
U40370_at	phosphodiesterase 1a, calmodulin-dependent	
Y00971_at	phosphoribosyl pyrophosphate synthetase 1	
X59618_at	ribonucleotide reductase m2 polypeptide	
Z46632_at	phosphodiesterase 4c, camp-specific	

**U94586_at**		

HT3620_s_at	fibroblast growth factor receptor 2	MAPK SIGNALING PATHWAY
M76559_at	calcium channel, voltage-dependent, alpha 2/delta subunit 1	
M38449_s_at	transforming growth factor, beta 1	
U66464_at	mitogen-activated protein kinase kinase kinase kinase 1	
U71087_at	mitogen-activated protein kinase kinase 5	
U13021_s_at	caspase 2, apoptosis-related cysteine peptidase (neural precursor cell expressed, developmentally down-regulated 2)	
D12625_at	neurofibromin 1	
S78187_at	cell division cycle 25b	

**X00274_at**		

M33600_f_at	major histocompatibility complex, class ii, dr beta 1	ANTIGEN PROCESSING AND PRESENTATION
X00274_at	major histocompatibility complex, class ii, dr alpha	
X03100_cds2	major histocompatibility complex, class ii, dp alpha 1	
M13560_s_at	cd74 antigen (invariant polypeptide of major histocompatibility complex, class ii antigen-associated)	
D45248_at	proteasome (prosome, macropain) activator subunit 2	
J00105_s_at	beta-2-microglobulin	
M17236_s_at	major histocompatibility complex, class ii, dq alpha 1	
HG658-HT658_f_at	major histocompatibility complex, class i, b	

**X69141_at**		

Y07512_at	protein kinase, cgmp-dependent, type i	GAP JUNCTION
M18255_cds2_s_at	protein kinase c, beta 1	
Z11695_at	mitogen-activated protein kinase 1	
X80763_s_at	5-hydroxytryptamine (serotonin) receptor 2c	

ANTIGEN PROCESSING AND PRESENTATION are processes that occur within a cell that result in fragmentation (proteolysis) of proteins, association of the fragments with MHC molecules, and expression of the peptide-MHC molecules at the cell surface where they can be recognized by the T cell receptor on a T cell. However, the path leading to the association of protein fragments with MHC molecules differs for class I and class II MHC. MHC class I molecules present degradation products derived from intracellular (endogenous) proteins in the cytosol. MHC class II molecules present fragments derived from extracellular (exogenous) proteins that are located in an intracellular compartment. Both MHC class I and class II pathways play an important role in anti-tumor immune responses. Patients with early intrahepatic recurrence of hepatocellular carcinoma (HCC) have the down-expressed expression both on MHC I and II pathways. It is generally acknowledged that tumors usually escape from host immune surveillance by dysfunction or defect of MHC I and MHC II presentation pathways with the down-expressed genes. Therefore, the down-expressed genes on the pathway may be one of the critical reasons for early intrahepatic recurrence. The causal relations among genes can also be identified in Figure 3. The down-expressed MHCI gene may cause the down-expressed *β*2m and the down-expressed MHCII and/or LI gene may cause the down-expressed SLIP and the down-expressed SLIP may cause the down-expressed CLIP. These causal relations may provide some implications on developing medicines against hepatocellular carcinoma. AXON GUIDANCE (also called axon pathfinding) is a subfield of neural development concerning the process by which neurons send out axons to reach the correct targets. Many axon guidance molecules may regulate cell migration and apoptosis in normal and tumorigenic tissues. Recent studies have shown that they are widely expressed outside the nervous system and that they may play important roles in HCC. Genes and their interactions are shown in Figure 4. For example, mutations in the ras oncogenes have been linked to many different cancers. Ras gene is over-expressed for HCC patients with early intrahepatic recurrence. The causal relations and gene-gene interactions are also shown in Figure 4. For instance, the down-expressed EpbB gene in patients with intrahepatic recurrence may cause the over-expressed ras gene through MAPK signaling pathway. The over-expressed DCC gene in patients with intrahepatic recurrence may cause the down-expressed Nck1 and over-expressed CALN, and so on. These gene-gene interactions may have prognostic implications for HCC.

### High-grade glioma data set [19]

50 high-grade glioma samples were carefully selected, 28 glioblastomas and 22 anaplastic oligodendrogliomas, all were primary tumors sampled before therapy. The classic subset of tumors were cases diagnosed similarly by all examining pathologists, and each case resembled typical depictions in standard textbooks. A total of 21 classic tumors was selected, and the remaining 29 samples were considered nonclassic tumors, lesions for which diagnosis might be controversial. Affymetrix arrays are used to determine the expression of over 12000 genes. The original goal is to separate the glioblastomas from the anaplastic oligodendrogliomas, which allows appropriate therapeutic decisions and prognostic estimation. The number of gene expression levels is 12625. Our goal is to identify genes and corresponding pathways associated with malignant gliomas.

**Figure 5:**
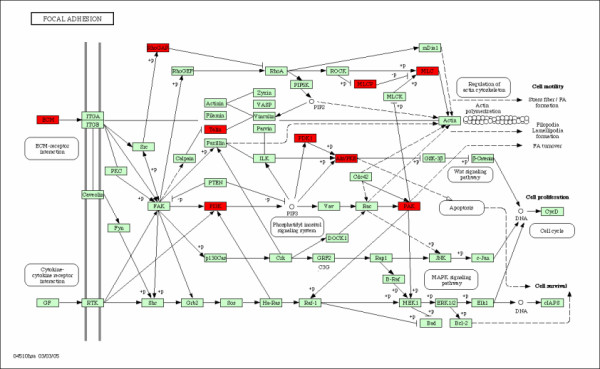
**FOCAL ADHESION**. FOCAL ADHESION pathway and the associated genes. The over-expressed and down-expressed genes on the pathway are shown in red and blue respectively.

**Figure 6:**
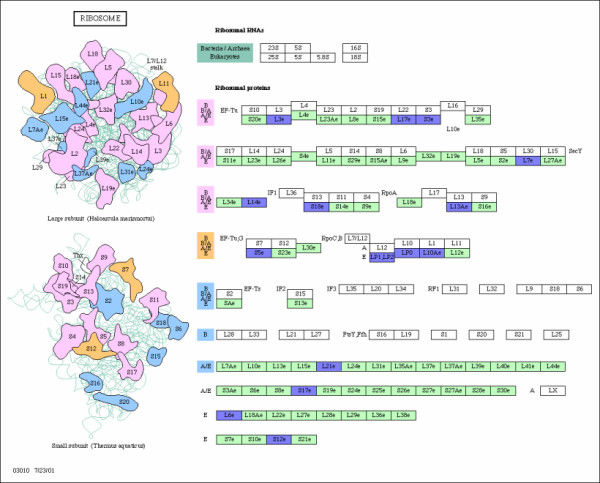
**RIBOSOME**. Ribosome pathway and the associated genes. The over-expressed and down-expressed genes on the pathway are shown in red and blue respectively.

BLpLog has identified 14 genes that are highly differentiated expressed in glioblastomas and anaplastic oligodendrogliomas (predicted AUC = 0.98). Eight pathways and associated correlated genes are identified with the integrated algorithm. The computational results are given in Table 5 and 6. The plots of FOCAL ADHESION and RIBOSOME pathways are shown in Figure 5 – Figure 6. The eight identified pathways are important in malignant gliomas and other diseases. For instance, COMPLEMENT AND COAGULATION CASCADES are composed of serine proteases that are activated through partial cleavage by an upstream enzyme. The elements of these cascades share several common structural characteristics, including a highly conserved catalytic site composed of Ser, His and Asp. The common principle underlying the organization of these systems is that proteases exist as inactive zymogens and are subsequently activated by upstream, active proteases. The initial activation might occur as a result of contact with a non-enzymatic ligand or cleavage by another protease. Understanding the interplay between complement and coagulation has fundamental clinical implications in the context of cancers with an inflammatory pathogenesis. Migration and invasion are important prerequisites for the infiltrative and destructive growth patterns of malignant gliomas. The glioma cell invasiveness depends on proteases of the coagulation and complement cascades. Another pathway, GLUTATHIONE METABOLISM, works through the operation of a group of enzymes called glutathione S-transferases (GST). Glutathione (GSH) plays a critical role in cellular mechanisms that result in cell death. The high glutathione levels may cause resistance to chemotherapy drugs. One interesting study by researchers in Texas showed that your chances of surviving a type of brain cancer, called primary malignant glioma, could depend on the type of glutathione-s-transferase (GST) gene you were born with. Therefore, it is possible to target glutathione metabolism in the prevention and treatment of malignant gliomas. Here we discuss the FOCAL ADHESION and RIBOSOME pathways in more details.

**Table 5:** BLpLog selected genes and the number of correlated

AFFY ID	Gene Description	# of corr Genes
yj12d03.s1 (+)	Soares placenta Nb2HP Homo sapiens cDNA clone IMAGE 148517 3	189
245_at(-)	HUMLNHR Human lymph node homing receptor mRNA, complete cds	149
32140_at (-)	H. sapiens mRNA for mosaic protein LR11	140
33117_r_at (-)	oq25a04.s1 Homo sapiens cDNA, 3 end	139
34279_at (+)	Homo sapiens mRNA	154
34645_at (-)	Human Hums3 mRNA for 40S ribosomal protein s3	76
35628_at (-)	Homo sapiens lamin B receptor homolog TM7SF2 mRNA, complete cds	191
36452_at (+)	Homo sapiens mRNA for KIAA1029 protein, complete cds	165
37591_at (-)	Human uncoupling protein homolog (UCPH) mRNA, complete cds	228
37915_at (+)	cDNA DKFZp434H071 (from clone DKFZp434H071)	290
38079_at (+)	cDNA DKFZp586B0918 (from clone DKFZp586B0918)	213
40419_at (-)	H. sapiens epb72 gene exon 1	204
41213_at (+)	H. sapiens mRNA for proliferation associated gene (pag)	158
675_at (-)	HUM927A Human interferon-inducible protein 9–27 mRNA, complete cds	210

**Table 6:** KEGG pathway and the highly correlated genes

Genes	Gene Description	Pathway
**yj12d03.s1 and 35628)_at**		

33520_at	coagulation factor vii (serum prothrombin conversion accelerator)	COMPLEMENT AND COAGULATION CASCADES
31591_s_at	complement factor h	
37550_at	coagulation factor viii, procoagulant component (hemophilia a)	
41701_at	complement component 6	
37175_at	serpin peptidase inhibitor, clade c (antithrombin), member 1	

**245_at and 36452_at**		

2023_g_at	v-akt murine thymoma viral oncogene homolog 2	FOCAL ADHESION
40438_at	protein phosphatase 1, regulatory (inhibitor) subunit 12a	
1560_g_at	p21 (cdkn1a)-activated kinase 2	
40162_s_at	cartilage oligomeric matrix protein	
33994_g_at	myosin, light polypeptide 6, alkali, smooth muscle and non-muscle	
33627_at	phosphoinositide-3-kinase, catalytic, delta polypeptide	
659_g_at	thrombospondin 2	
32029_at	3-phosphoinositide dependent protein kinase-1	
39765_at	talin 2	
41350_at	collagen, type vi, alpha 1	
954_s_at	protein phosphatase 1, catalytic subunit, alpha isoform	
38812_at	laminin, beta 2 (laminin s)	
37909_at	laminin, alpha 3	
34724_at	glucocorticoid receptor dna binding factor 1	
1557_at	p21/cdc42/rac1-activated kinase 1	

**32140_at**		

33134_at	adenylate cyclase 3	GAP JUNCTION
429_f_at	tubulin, beta 2b	
429_f_at	tubulin, beta 2a	
1000_at	mitogen-activated protein kinase 3	
429_f_at	tubulin, beta 4	

**33117_r_at and 34645_at**		

32440_at	ribosomal protein l17	RIBOSOME
32337_at	ribosomal protein l21	
31907_at	ribosomal protein l14	
36333_at	ribosomal protein l7	
31952_at	ribosomal protein l6	
32437_at	ribosomal protein s5	
35119_at	similar to ribosomal protein l13a	
31957_r_at	ribosomal protein, large, p1	
34645_at	ribosomal protein s3	
31538_at	ribosomal protein, large, p0	
31722_at	ribosomal protein l3	
31545_at	ribosomal protein s18	
32466_at	ribosomal protein l41	
34593_g_at	ribosomal protein s17	
35119_at	ribosomal protein l13a	
32394_s_at	ribosomal protein l23	
41178_at	ribosomal protein l11	
33117_r_at	ribosomal protein s12	
36786_at	ribosomal protein l10a	

**37591_at**		

37956_at	aldehyde dehydrogenase 3 family, member b2	METABOLISM OF XENOBIOTICS BY CYTOCHROME P45
375_at	glutathione s-transferase theta 1	
37707_i_at	alcohol dehydrogenase 5 (class iii), chi polypeptide	
33396_at	glutathione s-transferase pi	
1080_s_at	cytochrome p450, family 1, subfamily a, polypeptide 2	

**37915_at, 38079_at, and 675_at**		

36294_at	serine/threonine kinase 4	MAPK SIGNALING PATHWAY
39647_s_at	calcium channel, voltage-dependent, beta 2 subunit	
38743_f_at	v-raf-1 murine leukemia viral oncogene homolog 1	
39932_at	dual specificity phosphatase 7	
36935_at	ras p21 protein activator (gtpase activating protein) 1	
1292_at	dual specificity phosphatase 2	
34006_s_at	mitogen-activated protein kinase 8	
41432_at	ribosomal protein s6 kinase, 90 kda, polypeptide 5	
971_s_at	transforming growth factor, beta 2	
438_at	protein kinase, camp-dependent, catalytic, alpha	
1239_s_at	caspase 2, apoptosis-related cysteine peptidase	
38272_at	dual specificity phosphatase 14	
41226_at	dual specificity phosphatase 3	
37644_s_at	fas (tnf receptor superfamily, member 6)	
113_i_at	microtubule-associated protein tau	
249_at	nuclear factor of activated t-cells, cytoplasmic, calcineurin-dependent 4	
1788_s_at	dual specificity phosphatase 4	
31993_f_at	calcium channel, voltage-dependent, beta 2 subunit	
38079_at	guanine nucleotide binding protein (g protein), gamma 12	
1378_g_at	nuclear factor of kappa light polypeptide gene enhancer in b-cells 1 (p105)	
34521_at	mitogen-activated protein kinase kinase kinase 13	
37575_at	activating transcription factor 2	
1327_s_at	mitogen-activated protein kinase kinase kinase 5	
40030_at	protein kinase, y-linked	
36168_at	fibroblast growth factor receptor 1	
36004_at	inhibitor of kappa light polypeptide gene enhancer in b-cells, kinase gamma	
32304_at	protein kinase c, alpha	
39822_s_at	growth arrest and dna-damage-inducible, beta	
31618_at	tumor protein p53 (li-fraumeni syndrome)	
39672_at	protein tyrosine phosphatase, non-receptor type 7	
32749_s_at	filamin a, alpha (actin binding protein 280)	

**40419_at**		

40273_at	sphingosine kinase 2	GLYCOSPHINGOLIPID METABOLISM
41568_at	arylsulfatase d	
38818_at	serine palmitoyltransferase, long chain base subunit 1	
40214_at	udp-glucose ceramide glucosyltransferase	

**41213_at**		

556_s_at	glutathione s-transferase m4	GLUTATHIONE METABOLISM
556_s_at	glutathione s-transferase m1	
556_s_at	glutathione s-transferase m2 (muscle)	
32893_s_at	gamma-glutamyltransferase 2	
32893_s_at	gamma-glutamyltransferase 1	
32893_s_at	gamma-glutamyltransferase-like 4	

FOCAL ADHESIONS are large, dynamic protein complexes through which the cytoskeleton of a cell connects to the extracellular matrix, or ECM. They can be considered as sub-cellular macromolecules that mediate the regulatory effects (e.g. cell anchorage) of extracellular matrix (ECM) adhesion on cell behavior. Focal adhesions kinase (FAK) contributes to glioma growth and invasion. FAK integrates signals from activated growth factor receptors and integrins to regulate cell motility, invasion, proliferation, apoptosis, and angiogenesis. It, therefore, promotes tumor growth, and a role for FAK in glioma pathogenesis is suggested by its expression and localization. FAK genes are over-expressed on the pathway for glioblastoma patients as shown in Figure 5. These over-expressed genes may have many potential pro-tumorigenic functions and produce chemotherapy resistance in the glioblastoma patients. The causal relations and gene-gene interactions are also shown in Figure 5. The over-expressed ECM gene is a causal gene that causes several other genes to be over-expressed. If ECM is not present, the entire pathway is shut off. Conversely, the expression changes of downstream genes such as MLC and PAK may not affect the pathway as much.

Ribosome biology is huge and accounts for up to 50% of transcriptional activity of cells. The RIBOSOME pathway is composed of genes that encode various proteins of the ribosomal subunits. These proteins need to interact physically with each other to form a large protein complex, the ribosome, and are thereby closely related functionally. Ribosome biogenesis and translation are regulated at multiple levels and are associated with accurate cell growth and proliferation. Several tumor suppressors and proto-oncogenes have been found either to affect the formation of the mature ribosome or to regulate the activity of proteins known as translation factors. Disruption in one or more of the steps that control protein biosynthesis has been associated with alterations in the cell cycle and regulation of cell growth. Therefore, certain tumor suppressors and proto-oncogenes might regulate malignant progression by altering the protein synthesis machinery. The protein genes on RIBOSOME pathway (Figure 6) are down-expressed in glioblastoma patients. These down-expressed genes may provide some insight on how to develop new drugs and cancer therapies to target the RIBOSOME pathway involved in malignant gliomas.

## Conclusion

We have developed a Bayesian *L*_*p *_Logistic regression (BLpLog) method, defined a novel correlation measure, and proposed an integrated algorithm for gene selection and pathway identification. We have demonstrated that the simple Bayesian approach to integrating the regularization parameter out analytically performed well on prediction. The integrated algorithm can identify cancer associated genes and KEGG pathways efficiently with the test data sets. The correlation measure we defined can be used to hunt those upstream regularized genes with low expression levels, but are strongly correlated with the downstream highly differentiated genes identified with BLpLog. Almost all of the pathways found in this manuscript cannot be identified with the traditional correlation coefficient. The identified pathways can provide information on gene-gene interactions and causal relations for genes on the pathway. The knowledge of gene-gene interaction, gene regulation, and biological pathways can be applied to understanding the mechanisms of how pathway regulations have changed in different subtypes of cancer patients.

Mining high throughput data from different aspects can help us understand the cancer biology better. Our method provided much more information than we presented. For instance, we found hundreds of correlated genes for each downstream gene identified with BLpLog. Only a small proportion of the correlated genes is on the known KEGG pathways. We did not explore the functions and causal relations of the rest of the genes. Moreover, although there may be multiple pathways for each gene, we only reported the top pathway with the highest count of genes. We will infer the gene regulatory networks with the rest of the correlated genes and explore multiple pathways in the near future. Finally, gene set enrichment analysis [20] is a popular tool for evaluating microarray data at the level of gene sets. It, first, utilize a statistical test to identify highly differentiated upstream genes in two classes, and then define gene sets based on prior biological knowledge. There are two drawbacks with this method: (1) it is solely based on the partially known biological knowledge and (2) it cannot guarantee the upstream regularized genes to be selected in the set. It is, therefore, of great interests to incorporate the proposed correlation measure *R *into gene enrichment analysis, so that we can make sure the upstream regularized genes in the studied gene sets. This is the work of our future research.

## Methods

### *L*_*p *_Regularized Sparse Logistic Regression

A general binary classification problem may be simply described as follows. Given *n *samples, *D *= {(**x**_1_, *y*_1_), ..., (**x**_*n*_, *y*_*n*_)}, where **x**_*i *_is a multidimensional input vector with dimension *d *and class label *y*_*i *_∈ {-1, 1}, find a classifier *f*(**x**) such that for any input **x **with class label y, *f*(**x**) predicts class y correctly. The logistic regression is:

$$P(y=\pm 1|x,w)=g(yw^{T}x)=\frac{1}{1+\exp(-yw^{T}x)},$$

where **w **= (*w*_1_, ..., *w*_*d*_)^*T *^are the parameters which can be estimated through maximizing the log likelihood or minimizing the negative log likelihood.

$$E_{D}=-l(w|D)=\sum_{i=1}^{n}\log(1+\exp(-y_{i}w^{T}x_{i})).$$

Different prior assumptions of **w **in the maximum a-posteriori (MAP) estimation will lead to different regularization terms. The sparse parameter estimates can be achieved with *L*_*p *_regularization:

(1) *E *= *E*_*D *_+ *λL*_*p*_,

where

$$\begin{array}{ccc} L_{p}=\sum_{i=1}^{d}|w_{i}{|}^{p}, & \text{where} & 0<p\leq 1, \end{array}$$

where *λ *≥ 0 is a regularization parameter which must be tuned and $L_{p}=\sum_{j=0}^{d}|w_{j}{|}^{p}$ is the regularization term.

#### Bayesian Regularization

Choosing the best regularization parameter *λ *through cross-validation is time consuming. Therefore we propose a Bayesian regularization framework to integrate out the parameter following the similar methods of [11,21,22]. Minimizing E in equation (1) has the straight forward Bayesian interpretation. The posterior distribution for **w **is given by

(2) $$p(w|D)=\frac{p(D|w)p(w|\lambda )}{p(D)},$$

where *p*(*D*) = ∫ *P*(*D*|**w**)*p*(**w**|*λ*)d**w**d*λ *is a normalization factor that ensures that the posterior integrates to 1 and is given by an integral over the parameter space. The distribution of *p*(*D*|**w**) is defined as:

$$p(D|w)=\prod_{i=1}^{n}p(y_{i}|x,w)=\prod_{i=1}^{n}\frac{1}{\left(1+\exp(-y_{i}w^{T}x\right)}.$$

Let's $E_{w_{i}}=|w_{i}{|}^{p}$, and we have $L_{p}=\sum_{i=1}^{m}E_{w_{i}}$, where *m *is the number of non-zero model parameters and obviously *m *≤ *d*. The prior over model parameter, **w**, can be defined as

(3) $$p(w|\lambda )=\frac{1}{C(\lambda )}\prod_{i=1}^{m}\lambda \exp(-\lambda E_{w_{i}})=\frac{\lambda^{m}}{C(\lambda )}\exp(-\lambda L_{p}),$$

where $C(\lambda )=\int \prod_{i=1}^{m}\exp(-\lambda L_{p})\text{d}w$ is a normalization constant for a given *λ*. Taking the negative logarithm of the posterior density of equation (2), we have

- log *p*(**w**|*D*) = - log *P*(*D*|**w**) - log *p*(**w**|*λ*) + log *p*(*D*).

Therefore,

(4) - log *p*(**w**|*D*) = *E*_*D *_+ *λL*_*p *_+ log *p*(*D*) - *m *log *λ *+ log(*C*(*λ*)).

The hyperparameter *λ *can be integrated out analytically in the prior distribution *p*(**w**|*λ*).

*p*(**w**) = ∫ *p*(**w**|*λ*)*p*(*λ*)d*λ*.

As *λ *is a scaler, we can assign a new prior such that *p*(*λ*) ∝ *C*(*λ*)/*λ*. Substituting equation (3), we have

$$p(w)=\prod_{i=1}^{m}\int_{0}^{\infty }\lambda^{m-1}\exp\{-\lambda L_{p}\}\text{d}\lambda .$$

Using the Gamma integral $\int_{0}^{\infty }x^{v-1}e^{-\mu x}\text{d}x=\frac{\Gamma (v)}{\mu^{v}}$, we get

$$p(w)=\frac{\Gamma (m)}{L_{p}^{m}}.$$

Since $p(w|D)=\frac{P(D|w)p(w)}{p(D)}$, we have

(5) - log *p*(**w**|*D*) = *E*_*D *_+ *m *log *L*_*p *_+ log *p*(*D*) - log Γ(*m*).

Let *Q *= *E*_*D *_+ *m *log *L*_*p*_, comparing equation (4) with (5), we have

*E *= *E*_*D *_+ *λL*_*p *_= *E*_*D *_+ *m *log *L*_*p *_+ *R *= *Q *+ *R*,

where *R *= log *p*(*D*) - log Γ(*m*) is a constant not related to **w**. Therefore, *E *and *Q *are identical up to an additive constant and minimizing *E *is equivalent to minimizing the Bayesian regularization error function *Q*.

#### The BLpLog Algorithm

To find the optimal value of *Q *and **w**, we need to have the first and/or second order derivative with gradient based methods. One difficulty with *L*_*p *_is that it is not differentiable at zero and a differentiable approximation of *L*_*p *_has to be used. Differentiable approximations typically have a parameter that controls the trade-off between the smoothness of the approximation and the closeness of the non-differentiable function which is being approximated. One approximation which works for *p *≤ 1 is

$$L_{p}=\sum_{j=0}^{d}{\left(|w_{i}{|}^{2}+\gamma \right)}^{p/2},$$

where *γ *is the smoothing parameter. With this differentiable approximation, we get the following modified error function:

$$Q\approx Q_{\gamma }=E_{D}+m\log L_{p}=-l(w|D)+m\log(\sum_{j=0}^{d}{\left(|w_{i}{|}^{2}+\gamma \right)}^{p/2}).$$

Note that *Q*_*γ *_→ *Q *as *γ *→ 0.

Given a small value *γ*, the gradient can be calculated as:

(6) $$\nabla_{w}Q_{\gamma }=-\sum_{i}(1-g(y_{i}w^{T}x_{i}))y_{i}x_{i}+m\frac{\nabla_{w}L_{p}}{L_{p}},$$

where

$$\nabla_{w}L_{p}=\text{vec}\left\{\frac{pw_{i}}{{\left(|w_{i}{|}^{2}+\gamma \right)}^{1-p/2}}\right\},$$

where vec{·} represents a vector whose *i*-th element is given by the expression inside the brackets. The Hessian of the objective function is:

(7) $$H=\nabla_{ww}Q_{\gamma }=\sum_{i}g(w^{T}x_{i})(1-g(w^{T}x_{i}))x_{i}x_{i}^{T}+m\frac{L_{p}\nabla_{ww}L_{p}-{\left(\nabla_{w}L_{p}\right)}^{2}}{L_{p}^{2}},$$

where

$$\nabla_{ww}L_{p}=\text{diag}\left\{\frac{p}{{\left(|w_{i}{|}^{2}+\gamma \right)}^{1-p/2}}+\frac{p(p-2)|w_{i}{|}^{2}}{{\left(|w_{i}{|}^{2}+\gamma \right)}^{2-p/2}}\right\},$$

where diag{·} is the diagonal matrix whose *i*-th diagonal element is given by the expression inside the brackets. Let

*A *= diag{*g*(**w**^*T*^**x**_*i*_)(1 - *g*(**w**^*T*^**x**_*i*_))},

we have the matrix form of H:

(8) $$H=XAX^{T}+m\frac{L_{p}\nabla_{ww}L_{p}-{\left(\nabla_{w}L_{p}\right)}^{2}}{L_{p}^{2}}$$

With equation (6) and (8), we may estimate the parameters with Newton's method.

(9) **w**_*new *_= **w**_*old *_- *H*^-1^▽_**w**_*l*_*γ*_

We run the iteration until |**w**_*new *_- **w**_*old*_| <*δ*, where *δ *> 0 is a small number. In each iteration, *m *is not fixed, but updated as the number of |*w*_*i*_| > *β*, where *β *is small positive number. Other algorithms such as the fixed-Hessian or conjugate gradient may also be employed to solve the above problem. The advantage of Newton's method is that it converges very fast when near the optimal solution. This algorithm converges from any initialization and a local maximum is guaranteed.

There is no regularization parameter tuning in this algorithm, but we have to set several approximation parameters to some reasonable values to allow the algorithm to be performed well. Theoretically, *L*_*p *_penalty gives asymptotically unbiased estimates of the nonzero parameters while shrinking the estimates of zero (or small) parameters to zero when *p *→ 0 [16]. Unlike LASSO (with *p *= 1) that shrinks every parameter proportionally. Therefore the lower value of p would lead to more sparse and better solutions [12]. However when p is very close to zero, difficulties with convergence arise. Therefore, we set *p *= 0.1 in this paper. We also set the threshold *β *= 0.001. The smoothing parameter *γ *appears in the differentiable approximation to the *L*_*p *_norm. When *γ *is too large, the approximation is not a good one and the solution is overly smooth and the sparsity property of *L*_*p *_will be lost. When *γ *is very small, the number of iterations required for convergence increases drastically. We have found empirically that a choice of *γ *which does not require very many iterations, and yet converges to very sharp solutions is around 0.001–0.0000001 for our data We therefore set *γ *= 0.000001 in all experiments.

BLpLog employs a gradient decent method with superlinear convergence. To prevent the optimization from sticking to local optimal, we randomly initialize the coefficients 20 times and choose the estimated coefficients with the best AUC value for all of the computational experiments in this paper. Our experiments, however, have shown that the computational results are not sensitive to the parameter initialization and the algorithm converges quickly to the same optimization value most of the time with different parameter initializations.

Our binary classification algorithm can be extended for multiclass classification tasks. For a general c-class problem, we can employ the standard approach where two class classifiers are trained in order to separate each of the classes against all others. The decision rules are then coupled by voting, that is, sending the sample to the class with the largest probability.

### Similarity and Integrated Algorithm

In gene selection and pathway discovery, we have to deal with two important problems: (1) how to take into account the gene-gene correlations and (2) how to hunt the upstream regulatory genes. As we discussed in the previous section, current gene selection methods can only select the downstream genes with bigger changes in expression. The characteristic of the regulatory genes is that their gene expression changes may be low but they are highly correlated with the downstream highly expressed genes. We first introduce our own correlation (similarity) measure (R) for continuous variables such as gene expression data.

$$R(x,y)=\frac{cov(x,y)}{\min\{var(x),var(y)\}},$$

where $cov(x,y)=\sum (x_{i}-\bar{x}){\left(y_{i}-\bar{y}\right)}^{T}$ is the standard covariance and $var(x)=\sum (x_{i}-\bar{x}){\left(x_{i}-\bar{x}\right)}^{T}$ is the variance. Based on this definition we have *R*(**x**, **y**) = *R*(**y**, **x**), and *R *= 0 when **x **and **y **are independent and *R *≥ 1, when either *var*(**x**) ≤ *cov*(**x**, **y**) or *var*(**y**) ≤ *cov*(**x**, **y**). It is clear that this definition of R is different from the standard correlation coefficient $r=cov(x,y)/\sqrt{var(x)var(y)}$ in its denominator. However, R can catch the the genes that have very small changes in expression but are highly correlated with the significantly expressed (downstream) genes. For instance, given *cov*(**x**, **y**) = 0.01, *var*(**x**) = 0.01, and *var*(**y**) = 1, we have both *R *= 1 and *r *= 0.1. Our definition of R guarantees that the upstream regulatory genes and the downstream genes will be in the same group.

### The Integrated Algorithm

We now incorporate the correlation structure into the gene selection and pathway identification algorithm. Given a set of n independent observations ${\left\{x_{i},y_{i}\right\}}_{i=1}^{n}$:

1. The gene selection step: Identifying a small subset of individual genes that are associated with cancer using *L*_*p *_regularized Bayesian logistic regression

2. For each selected gene **x**_*i*_, find all **x**_*j *_from the original data set, such that |*R*(**x**_*i*_, **x**_*j*_)| ≥ *h*, where *h *is a threshold and set to 0.9 for experiments in this paper.

3. For each subset of selected genes, identify the pathways associated with it using DAVID.

## Authors' contributions

ZL conceptualized and designed method, developed the software, and wrote the manuscript. RG and FJ analyzed and interpreted the data on its biological contents. MT helped in method design and manuscript writing and revised the manuscript critically. XJ did the actual computations. All authors read and approved the final manuscript.
